# Role and interpretation of FDG-PET/CT in HIV patients with fever of unknown origin: a prospective study

**DOI:** 10.7448/IAS.15.6.18107

**Published:** 2012-11-11

**Authors:** C Martin, C Castaigne, M Tondeur, P Flamen, S De Wit

**Affiliations:** 1 CHU Saint-Pierre, Department of Infectious Diseases, Brussels, Belgium; 2 CHU Saint-Pierre, Department of Nuclear Medicine, Brussels, Belgium

## Abstract

**Purpose of the study:**

Fever of unknown origin (FUO) is a challenging clinical entity in HIV patients. FDG-PET/CT is well validated in the work-up of FUO in HIV-negative patients but in HIV viremic patients, metabolism of HIV reactive lymph nodes could decrease its specificity. We prospectively evaluated the usefulness of FDG-PET/CT in FUO in HIV-positive patients and in particular whether HIV viremia impacts on FDG-PET/CT performance.

**Methods:**

FDG-PET/CT was performed in 20 HIV patients with FUO and compared with FDG-PET/CT in 10 HIV viremic patients without FUO. Final diagnosis for FUO was based on histopathology, microbiology, or clinical and imaging follow-up. Mode of diagnosis, accordance of FDG-PET/CT with final diagnosis, localization of invasive diagnosis procedures was recorded in order to assess usefulness of FDG-PET/CT.

**Results:**

FDG-PET/CT showed a different pattern in FUO and asymptomatic viremic patients. Reactive HIV lymph nodes in asymptomatic viremic patients were mostly peripheral with mean SUVmax of 6.5. In patients with FUO and underlying focal pathologies, hypermetabolic lymph nodes were central with mean SUVmax of 11.6. Presence of central lymph nodes with high FDG uptake in had a 100% specificity for focal pathology, even in viremic patients and absence of these had 100% negative predictive value. Lymph node biopsy in central hypermetabolic areas allowed identifying underlying disease in all FUO patients. For peripheral lymph nodes, a ROC curve was built in order to define the best cut-off of SUVmax for biopsy: SUVmax of 6–8 showed a sensitivity of 62.5% and specificity of 75%. Lymph nodes with SUVmax<4 had sensitivity of 0%.

**Conclusions:**

FDG-PET/CT contributed to the diagnosis or exclusion of a focal etiology of the febrile state in 80% of HIV patients with FUO. Although number of patients was small, we could highlight several clear-cut features to help interpreting FDG-PET/CT in HIV patients with FUO. As in HIV-negative patients, we showed the usefulness of FDG-PET/CT in FUO in HIV patients even if they are viremic.

